# Draft Genome Sequence of the Cyanobacterium Aphanizomenon flos-aquae Strain 2012/KM1/D3, Isolated from the Curonian Lagoon (Baltic Sea)

**DOI:** 10.1128/genomeA.01392-14

**Published:** 2015-01-15

**Authors:** Sigitas Šulčius, Gediminas Alzbutas, Kotryna Kvederavičiūtė, Judita Koreivienė, Linas Zakrys, Arvydas Lubys, Ričardas Paškauskas

**Affiliations:** a Marine Science and Technology Center, Klaipėda University, Klaipėda, Lithuania; b Thermo Fisher Scientific Baltics, Vilnius, Lithuania; c Institute of Biotechnology, Vilnius University, Vilnius, Lithuania; d Institute of Botany, Nature Research Centre, Vilnius, Lithuania

## Abstract

We report here the *de novo* genome assembly of a cyanobacterium, Aphanizomenon flos-aquae strain 2012/KM1/D3, a harmful bloom-forming species in temperate aquatic ecosystems. The genome is 5.7 Mb with a G+C content of 38.2%, and it is enriched mostly with genes involved in amino acid and carbohydrate metabolism.

## GENOME ANNOUNCEMENT

The filamentous and nitrogen-fixing cyanobacterium Aphanizomenon flos-aquae is globally distributed in temperate lakes and brackish water ecosystems, among other locations in the Curonian Lagoon of the Baltic Sea (1). Blooms of A. flos-aquae affect water quality and the recreational value of the ecosystem, and they endanger aquatic biota, animal health, and human health (2–4). There is presently only one draft genome sequence available for this species (5); yet, little is known about the genetic capabilities of this cyanobacterium.

The clonal Aphanizomenon flos-aquae strain 2012/KM1/D3 was isolated from the Curonian Lagoon during the bloom in 2012 and is maintained as a unicyanobacterial yet nonaxenic culture. DNA was extracted using a cetyltrimethylammonium bromide (CTAB) protocol (6), with some modifications. Cell lysis was facilitated by five freeze (in liquid nitrogen)/thaw (at 65°C) cycles following treatment with lysozyme (50 mg/ml), RNase (100 mg/ml), proteinase K (20 mg/ml), and SDS (10%). The draft genome sequence was performed by Thermo Fisher Scientific Baltics (http://www.thermofisher.lt) using the Personal Genome Machine (PGM) with the Ion PGM sequencing 400 kit and Thermo Scientific MuSeek library preparation kit (catalog no. 4480829). A total of 651 Mb of DNA sequence and 2,531,067 reads were generated. To filter out cyanobacterial reads and remove sequences belonging to associated bacteria (7), phylogenetic classification of all reads was done using the Kraken (8) and PhymmBL (9) programs. Genome assembly was carried out by SeqMan (DNAStar Lasergene version 11.1.0; Madison, WI, USA), SPAdes version 3.1.1 (10), and MIRA version 4.0.2 (11), and the final genome assembly was created by merging the results using CISA (12). Genome annotation was carried out using RAST (13) and the NCBI Prokaryotic Genome Annotation Pipeline (PGAP). The genome was validated with QUAST (14).

The A. flos-aquae draft genome is 5,741,771 bp distributed in 325 contigs, with an average G+C content of 38.2% and an *N*_50_ value of 25,535. The largest contig is 120,451 bp. PGAP identified 5,478 protein-coding genes, of which 4,415 had a predicted function and 1,010 are pseudogenes. The genome contains 52 RNA genes, of which 38 are tRNA and 14 are rRNA genes. The calculation of the average nucleotide identity (ANI) based on the BLAST algorithm showed that the closest sequences match to *A*. *flos-aquae* strain NIES-81 (5) at 96.24% similarity. These results corresponded well to the tetranucleotide signature frequency correlation coefficient (0.997), which indicates the taxonomic relatedness of two strains (15). The genome contains fragments of cyanotoxin gene clusters similar to those observed for other Aphanizomenon strains (16) and possesses a high number of clustered regularly interspaced short palindromic repeats (17). The genome also contains genes involved in the metabolism of glycine betaine, which has been shown to be a very efficient osmolyte under conditions of saline stress, which occurs often in transitional aquatic ecosystems. This study will further facilitate our understanding of the genomic capabilities of A. flos-aquae to interact with the surrounding environment.

### Nucleotide sequence and accession numbers.

This whole-genome shotgun project has been deposited at DDBJ/EMBL/GenBank under the accession no. JSDP00000000. The version described in this paper is version JSDP01000000.
